# Meningococcal Disease Surveillance in Men Who Have Sex with Men — United States, 2015–2016

**DOI:** 10.15585/mmwr.mm6738a4

**Published:** 2018-09-28

**Authors:** Catherine H. Bozio, Amy Blain, Jessica MacNeil, Adam Retchless, Lauren M. Weil, Xin Wang, Laurel T. Jenkins, Lorraine D. Rodriguez-Rivera, Claire Jarashow, Van Ngo, Susan Hariri, Sarah A. Mbaeyi, Sara Oliver

**Affiliations:** ^1^Division of Bacterial Diseases, National Center for Immunization and Respiratory Diseases, CDC; ^2^Epidemic Intelligence Service, CDC; ^3^Los Angeles County Department of Public Health, California.

Meningococcal disease is a rare, but serious, bacterial infection that progresses rapidly and can be life-threatening, even with prompt antibiotic treatment. Men who have sex with men (MSM) have previously been reported to be at increased risk for meningococcal disease compared with other men, and recent outbreaks of serogroup C meningococcal disease among MSM have occurred (*1*). However, the epidemiology of meningococcal disease among MSM in the United States is not well described, in part, because information about MSM has not historically been collected as part of routine meningococcal disease surveillance. To better characterize and identify risk factors for meningococcal disease in general, supplementary data and isolates have been collected since 2015 through enhanced meningococcal disease surveillance activities. During 2015–2016, 271 cases of meningococcal disease in men aged ≥18 years were reported to the National Notifiable Diseases Surveillance System (NNDSS) in 45 states participating in this enhanced surveillance. Forty-eight (17.7%) cases were in men identified as MSM, including 17 (37.8%) with human immunodeficiency virus (HIV) infection. Among MSM, 39 (84.8%) cases were caused by *Neisseria meningitidis* serogroup C, whereas this serogroup was responsible for only 16.4% of cases among men who were not known to be MSM (non-MSM). Despite improvements in surveillance, MSM likely remain underascertained among men with meningococcal disease. Improved surveillance data are needed to understand the prevalence of and risk for meningococcal disease among MSM and inform policy and prevention strategies. Vaccination with quadrivalent meningococcal conjugate (MenACWY) vaccine is recommended for the control of meningococcal disease outbreaks caused by serogroups A, C, W, or Y, including during outbreaks among MSM; in addition, all persons aged ≥2 months with HIV infection should receive MenACWY vaccine because of the increased risk for meningococcal disease.

Since 2003, seven outbreaks (*2*) of serogroup C meningococcal disease have been reported among MSM in four metropolitan areas in the United States (Chicago, Los Angeles County/Southern California, Miami, and New York City) (*1*,*3*). An analysis of cases reported in NNDSS during January 2012–June 2015 demonstrated that the risk for meningococcal disease among MSM (0.56 cases per 100,000 population) was four times that among other men (0.14) (*1*). Whereas HIV infection appeared to be associated with the increased risk for sporadic illness observed in that study, additional risk factors for meningococcal disease, including in outbreak-associated cases, have not been well established. The prevalence and epidemiology of meningococcal disease in this population remain poorly described because information to identify MSM and HIV infection was not routinely collected before 2015.

In 2015, enhanced meningococcal disease surveillance activities were implemented in 45 U.S. states as part of the Epidemiology and Laboratory Capacity for Infectious Diseases Cooperative Agreement to routinely collect isolates and supplementary data (including information to identify MSM and HIV infection) on meningococcal disease cases reported to NNDSS. To assess completeness of this information and report updated findings on MSM with meningococcal disease, all confirmed and probable meningococcal disease cases among men aged ≥18 years reported to NNDSS by enhanced meningococcal disease surveillance–participating states during January 2015–December 2016 were reviewed. State or local health departments classified cases as occurring in either MSM or non-MSM; the latter group included men for whom information to identify MSM was missing. During this 2-year period, 39 state health departments identified MSM by asking adult male patients about either their sexual orientation or gender of their main sex partner or both or by obtaining this information from other sources (e.g., medical record); six states did not collect this information. States also classified cases as occurring in men with and without HIV infection, as well as being outbreak-associated or sporadic. A case report form to collect additional data on potential risk factors was completed for cases occurring in MSM. Serogroup was determined by polymerase chain reaction and slide agglutination. Sequence type (ST) was determined using whole genome sequencing. Incidence was calculated as the number of meningococcal disease cases per 100,000 men aged ≥18 years. Population denominators for non-MSM (*4*) and MSM (*5*) were derived from the 2016 American Community Survey. HIV prevalence among MSM was estimated from the 2014 National HIV Behavioral Surveillance Report (*6*).

During 2015–2016, a total of 271 cases of meningococcal disease in men aged ≥18 years were reported. Among these, sufficient information to identify MSM was available for 124 (45.8%). Overall, 48 (17.7%) cases occurred in MSM (Table 1). Information on HIV status was available for 133 (49.1%) cases, although completeness of this information was higher among MSM (45 of 48; 93.8%) than among non-MSM (89 of 223; 39.5%). Among the 133 men with known HIV status, 17 (12.8%) had HIV infection, all of whom were MSM, accounting for 37.8% of 45 MSM with known HIV status. Among cases in MSM, the median age was 32 years, 66.7% of patients were white, and 77.8% were non-Hispanic. In contrast, among 223 cases in non-MSM, the median age was 41 years, 68.3% were white, and 81.7% were non-Hispanic. All cases in MSM were reported from 12 states. Thirty-two (66.7%) cases in MSM were associated with three outbreaks, which were reported through enhanced surveillance activities: 11 cases in the Chicago outbreak, 20 cases in the Southern California outbreak, and one case in Miami (four additional outbreak-associated cases were reported in 2017). Six (12.5%) additional cases were reported from jurisdictions that had previously reported an outbreak of meningococcal disease. In contrast, 18 (8.1%) cases in non-MSM were associated with an outbreak of meningococcal disease. *N. meningitidis* serogroup C accounted for 39 (84.8%) cases in MSM, compared with 16.4% of cases in non-MSM (Table 1). Among cases of serogroup C meningococcal disease in MSM with available molecular data, all were caused by ST-11 strains, although the meningococci had five different molecular profiles, as defined by the combination of ST, FetA, PorA, and PorB types. The outbreaks in Chicago and Southern California involved meningococci with different molecular profiles, and thus were distinct from each other. Among MSM, six of 48 (12.5%) cases were fatal, whereas 30 of the 203 (14.8%) cases in non-MSM were fatal. The case-fatality ratio was not statistically significantly different in MSM with and without HIV infection (11.8% and 14.3%, respectively). Among cases in MSM, HIV infection status and case-fatality ratios were not statistically significantly different among outbreak-associated cases (35.5% and 12.5%, respectively) and sporadic cases (42.9% and 12.5% respectively).

**TABLE 1:** Characteristics of meningococcal disease cases among men aged ≥18 years, by MSM status — United States, 2015–2016

Characteristic	MSM (n = 48)		Non-MSM* (n = 223)	
	No. (%)^†^	% Completeness^§^	No. (%)^†^	% Completeness^§^
**Age group (yrs)**				
18–24	6 (12.5)	100.0	50 (22.4)	100.0
25–29	12 (25.0)		20 (9.0)	
30–39	15 (31.3)		36 (16.1)	
40–49	6 (12.5)		26 (11.7)	
50–64	6 (12.5)		63 (28.3)	
≥65	3 (6.3)		28 (12.6)	
**Total**	**48**		**223**	
**Race**				
White	30 (66.7)	93.8	129 (68.3)	84.8
Black	13 (28.9)		53 (28.0)	
Other^¶^	2 (4.4)		7 (3.7)	
**Total**	**45**		**189**	
**Ethnicity**				
Hispanic	10 (22.2)	93.8	34 (18.3)	83.4
Non-Hispanic	35 (77.8)		152 (81.7)	
**Total**	**45**		**186**	
**HIV infection status**				
Infected	17 (37.8)	93.8	0 (—)	39.5
Uninfected	28 (62.2)		88 (100.0)	
**Total**	**45**		**88**	
**Associated with MD outbreak**				
Yes	32 (66.7)	100.0	18 (8.1)	100.0
No	16 (33.3)		205 (91.9)	
**Total**	**48**		**223**	
**Outcome**				
Survived	42 (87.5)	100.0	173 (85.2)	91.0
Died	6 (12.5)		30 (14.8)	
**Total**	**48**		**203**	
**Serogroup**				
B	4 (8.7)	95.8	81 (40.3)	90.1
C	39 (84.8)		33 (16.4)	
W	1 (2.2)		26 (12.9)	
Y	1 (2.2)		38 (18.9)	
Nongroupable	1 (2.2)		22 (10.9)	
**Total**	**46**		**201**	

**Abbreviations:** HIV = human immunodeficiency virus; MD = meningococcal disease; MSM = men who have sex with men.

* Includes 76 men known to be non-MSM and 147 men with missing MSM information.

^†^ Calculated among those with a known response.

^§^ Number of known responses divided by the total number of responses.

^¶^ Including Asian and other race.

The incidence of meningococcal disease among MSM was 0.54 cases per 100,000 population (Table 2). The incidence of meningococcal disease among MSM in jurisdictions that reported an outbreak of meningococcal disease among MSM was 3.27 cases per 100,000 population, which was higher than the rate in jurisdictions that did not report an outbreak of meningococcal disease (0.19 cases per 100,000). The incidence of reported cases among non-MSM men was 0.10 per 100,000.

**TABLE 2:** Incidence of reported meningococcal disease among men who have sex with men (MSM) and men not known to be MSM (non-MSM) aged ≥18 years — United States, 2015–2016

Category	No. of cases	Estimated population	Incidence (per 100,000)
**Non-MSM, all**	223	224,572,168	0.10
**MSM, all**	48	8,879,801	0.54
MSM, outbreak*	32	979,522	3.27
HIV-infected^†^	11	202,761	5.43
HIV-uninfected^†^	20	776,761	2.57
**MSM, sporadic^§^**	15	7,900,279	0.19
HIV-infected^¶^	5	1,706,460	0.29
HIV-uninfected^¶^	8	6,193,819	0.13

**Abbreviation:** HIV = human immunodeficiency virus.

* Cases reported in jurisdictions that reported a cluster or outbreak of meningococcal disease among MSM; denominator was estimated from the population in the counties within these jurisdictions.

^†^ HIV status was available for 31 of 32 cases.

^§^ Cases reported in jurisdictions that did not report a cluster or outbreak of meningococcal disease among MSM; denominator was estimated from the population in the counties within these jurisdictions.

^¶^ HIV status was available for 13 of 15 cases.

## Discussion

Meningococcal disease incidence has been decreasing in all age groups in the United States since 1996 (*7*), although outbreaks continue to occur. The results of this analysis, using data collected through enhanced meningococcal disease surveillance activities, are consistent with a previous report (*1*) demonstrating that the increased incidence of reported meningococcal disease among MSM is largely driven by outbreaks. In the nonoutbreak setting, HIV appears to be a likely risk factor for disease; the incidence of sporadic meningococcal disease was higher in HIV-infected MSM compared to HIV-uninfected MSM. The role of other potential risk factors remains unclear, highlighting the need to strengthen surveillance and collect additional data.

Identifying MSM among meningococcal disease patients and improving collection of data on HIV status will be important to better understand the epidemiology and risk factors for transmission and disease among MSM and to guide meningococcal vaccination policy and other prevention strategies. Because persons with HIV infection have an increased risk for meningococcal disease, the Advisory Committee on Immunization Practices recommends that persons aged ≥2 months with HIV infection receive MenACWY vaccine (*8*); currently, no recommendation exists for routine vaccination with meningococcal conjugate vaccine for all MSM.

The findings in this report are subject to at least two limitations. First, half of meningococcal disease cases among adult men did not have information allowing identification of MSM, and thus were presumed to have occurred among non-MSM for this analysis, reflecting the likely underascertainment and potential misclassification of some cases among MSM as cases among non-MSM. However, no standard surveillance definition for MSM currently exists, despite MSM being at increased risk for other infectious diseases (*9*). In addition, because 79% of cases in MSM were identified in jurisdictions that had ever reported an outbreak of meningococcal disease, it is unclear whether this high proportion reflects the actual epidemiology in this population or whether ascertainment is better in these jurisdictions as a consequence of heightened awareness because of past outbreaks. Second, whereas completeness of data on HIV status was high among cases in MSM, improved completeness of HIV status among men who were not known to be MSM is important for understanding the role of HIV infection in the risk for meningococcal disease among MSM and the general population. A higher proportion of cases in MSM had HIV infection, although the low completeness of data on HIV status of cases among non-MSM men limits the ability to accurately describe the proportion with HIV infection.

Although enhanced meningococcal disease surveillance fills an important gap in meningococcal disease surveillance, the limitations of this analysis reflect areas for strengthening surveillance. In addition, vaccination with MenACWY vaccine is recommended for the control of meningococcal disease outbreaks due to serogroups A, C, W, or Y, including during outbreaks among MSM; in addition, all persons aged ≥2 months with HIV infection should receive MenACWY vaccine because of the increased risk of meningococcal disease (*8*). During investigations of meningococcal disease caused by any serogroup, state and local health departments are encouraged to assess HIV status of all patients and identify MSM among male patients aged ≥16 years.* All state health departments are asked to submit any available isolates to CDC for whole genome sequencing.

**Summary**

**What is already known about this topic?**

Men who have sex with men (MSM) have been reported to be at increased risk for meningococcal disease in the United States. The epidemiology of disease in this group is not well described because information on MSM historically has not been collected through routine meningococcal disease surveillance.

**What is added by this report?**

Enhanced surveillance demonstrates that MSM, including those with human immunodeficiency virus (HIV) infection, have an increased meningococcal disease incidence compared with that in non-MSM.

**What are the implications for public health practice?**

Identifying MSM among meningococcal disease patients and improving collection of data on HIV status for all cases are important to understanding the epidemiology and risk factors for meningococcal disease among MSM.

## Footnotes

* As part of enhanced surveillance activities, state and local health departments are encouraged to identify MSM among male patients aged ≥16 years. The analysis was restricted to those aged ≥18 years to be consistent with previous analyses and to calculate incidence using available denominator data.
